# Neuroprotective Effect of Ethanol Extract of Leaves of *Malva parviflora* against Amyloid-*β*- (A*β*-) Mediated Alzheimer's Disease

**DOI:** 10.1155/2014/156976

**Published:** 2014-10-29

**Authors:** Muhammad Aslam, Ali Akbar Sial

**Affiliations:** ^1^Department of Basic Medical Sciences, Faculty of Pharmacy, Ziauddin University, Karachi 75600, Pakistan; ^2^Department of Pharmaceutics, Faculty of Pharmacy, Ziauddin University, Karachi 75600, Pakistan

## Abstract

*Malva parviflora* L. possesses significant antioxidant potential. This study was conducted to evaluate the neuroprotective effect of ethanol extract of the leaves of *Malva parviflora* against amyloid-*β*- (A*β*-) mediated Alzheimer's disease. In Morris water maze model, the extract significantly restored the defected memory of amyloid-*β* injected mice (*P* < 0.01). The reduced levels of brain antioxidant enzymes such as glutathione peroxidase, glutathione reductase, catalase, and superoxide dismutase were also restored significantly to similar levels as seen in normal control mice (*P* < 0.01). The levels of lipid peroxidase were decreased significantly in treatment group mice when compared to Alzheimer group mice (*P* < 0.01). So, this study showed that ethanol extract of the leaves of *Malva parviflora* possesses neuroprotective activity in mice.

## 1. Introduction

Alzheimer's disease (AD) is a progressive neurodegenerative disease. World Health Organization (WHO) has reported that 5% of males and 6% of females of age 60 years or above are affected with Alzheimer's type dementia worldwide [1]. Oxidative stress has been a major cause of neurotoxicity in Alzheimer's disease and other neurodegenerative disorders. Amyloid-*β* (A*β*) causes oxidative damage in brain which may lead to Alzheimer's disease [2].

*Malva parviflora *L. (cheeseweed) family Malvaceae is a wonderful gift from nature for mankind. This plant has shown its pharmacological potential in different ailments. Leaves are used in the management of wounds and swelling. A lotion made from the leaves is used to treat bruises and broken limbs [3]. The leaves of* M. parviflora *are used for drawing swollen, inflamed purulent wounds [4]. Pharmacological studies show that* Malva parviflora* possesses antibacterial [5], antidiabetic [6], antifungal [7], and other activities.

It is well known that plants have been a major source of natural antioxidants [8].* Malva parviflora *is one of suchplants. The plant contains flavonoids and phenolic compounds [9].* Malva parviflora *has shown significant antioxidant potential [10].

Based on the notion that antioxidant herbs and food are effective in the management of neurodegenerative diseases, we evaluated the neuroprotective effect of ethanol extract of the leaves of* Malva parviflora *in this study.

## 2. Materials and Methods

### 2.1. The Collection of Plant Material

Fresh leaves of* Malva parviflora* L. were collected from Khairpur, Sindh, Pakistan. A pharmacognosist of the Department of Pharmacognosy, Federal Urdu University, Pakistan, authenticated the sample. Voucher specimen (RP/PHARM/1390) was deposited in the institute for future reference.

### 2.2. Preparation of Ethanol Extract

The leaves of* Malva parviflora* were shade-exsiccated under room temperature. Fine powder was prepared from 500 grams of the leaves. The powder was soaked in ethanol for one hour. Now, the powdered material was extracted using a percolator for 72 hours. In the subsequent step, the extract was filtered and made solvent-free using a rotary evaporator. In the final step, the extract was further freeze-dried to produce a dry powder [11].

### 2.3. Drugs and Chemicals

Amyloid-*β* protein fragment 25–35 (A4559 SIGMA) and ethanol (46139 FLUKA) were purchased from Sigma-Aldrich.

### 2.4. The Selection of Animals

*Swiss albino *mice weighing between 25 and 30 g were used in this study. The specifications given in Helsinki Resolution 1964 were followed during animal handling. This research was approved by our institutional ethical committee (number 11/PHA/210).

## 3. Determination of Acute Oral Toxicity

Acute toxicity of ethanol extract of* Malva parviflora* leaves was evaluated at a dose range of 250–1000 mg/kg using Litchfield and Wilcoxon method. No signs of any toxicity were observed at this dose range [12].

### 3.1. Dosing

The dose of the extract was calculated according to the body weight of the mice. The extract was given at two different doses, namely, 250 mg/kg and 500 mg/kg. The dosing of the extract was done daily in normal doses according to the body weight of the animals [13].

### 3.2. Methodology

A total number of 40 healthy* Swiss albino *mice weighing between 25 and 30 g were procured from the animal house of University of Karachi, Pakistan. Six animals were kept per cage in polypropylene cages with a layer of sawdust litter under controlled conditions at room temperature 25–30°C, relative humidity 45–55%, and 12/12 hours light-dark cycle. The mice were given standard pellets and water* ad libitum*. The pellets and water were withdrawn six hours before the administration and during the experiments. The mice were divided into four groups, namely,

 Group I: Normal control, given normal saline 1 mL/kg;
 Group II: Alzheimer group, given amyloid-*β* (A*β*) protein 10 *μ*L/animal;
 Group III: Treatment group, given amyloid-*β* (A*β*) protein 10 *μ*L/animal and ethanol extract at the dose of 250 mg/kg;
 Group IV: Treatment group, given amyloid-*β* (A*β*) protein 10 *μ*L/animal and ethanol extract at the dose of 500 mg/kg.

The extract was administered to all animals by oral gavage once a day for 21 days prior to amyloid-*β* protein injection and continued for a further 7 days.

### 3.3. Intracerebroventricular (ICV) Injection of Amyloid-*β* Protein Fragment (25–35)

Amyloid-*β* (25–35) protein mixed with normal saline and incubated for 4 days at 37°C was given through intracerebroventricular (ICV) injections to induce Alzheimer's disease in Groups II, III, and IV on day 21. In Group I, control group, mice were given normal saline by intracerebroventricular injection route. A*β* (25–35) was administered at the bregma point with a 50 *μ*L Hamilton microsyringe using a 26-gauge needle that was inserted 2.4 mm deep. In other words, the needle was inserted unilaterally 1 mm to the right of the middle point equidistant from each eye slightly angled towards 45° perpendicular to the plane of the skull. Mice showed normal behaviour within 1 min after injection and the injection volume was 10 *μ*L/mouse [14].

### 3.4. Effect of the Extract on Memory of Mice

For the assessment of the effect of the extract on memory of mice, Morris water maze test was used. The test was conducted as per the procedure and the parameters described earlier [15]. The scored parameters were escape latency (EL), the time taken by the animal to move from the starting quadrant to find the hidden platform in the target quadrant, and time spent in the target quadrant (TSTQ) [16].

### 3.5. Effect of the Extract on Brain Enzymes

The mice were sacrificed by cervical dislocation on the 29th day of the study. The brains of mice were dissected out. Biochemical assay was performed after washing the brains with ice-cold normal saline. At first, the tissues were homogenized in Tris HCl and then centrifuged for 10 min at 10,000 ×g at 4°C. For the assessment of neuroprotective activity of the extract, the concentrations of antioxidant enzymes were estimated. Calorimetric techniques were used in the estimation of enzymes as per standard procedures for glutathione peroxidase (GSHx) [17], glutathione reductase (GSHr) [18], catalase (CAT) [19], lipid peroxidation (LPO) [20], and superoxide dismutase (SOD) [21].

### 3.6. Statistical Analysis

The data expressed are mean ± standard error of mean (SEM) with 95% confidence intervals (CI). Data were analysed by one-way ANOVA followed by Tukey's post hoc test. All statistical analyses were carried out by using Graph Pad Prism version 5.00 for Windows, Graph Pad Software, San Diego, CA, USA. A probability level of 0.01 or less was accepted as significant.

## 4. Results

**Figure 1:**
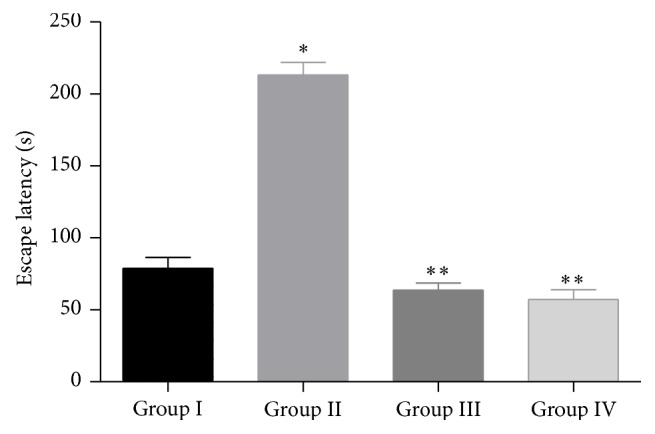
Effect of ethanol extract of leaves of* Malva parviflora* on escape latency (EL) in Morris water maze test. The values are mean ± SEM. *n* = 10. ^*^ *P* < 0.01, significant difference when compared to normal control mice. ^**^ *P* < 0.01, significant difference when compared to Alzheimer group mice (One-way ANOVA followed by Tukey's post hoc test).

The results of the Morris water maze test show that there was a significant increase in escape latency (EL) of Group II (Alzheimer group) mice when compared to Group I (Normal control) mice (*P* < 0.01). However, the time taken by the mice to move from the starting quadrant to find the hidden platform in the target quadrant (EL) was decreased significantly in Group III (Treatment group; 250 mg/kg) and Group IV (Treatment group; 500 mg/kg) mice when compared to Group II (Alzheimer group) mice (*P* < 0.01) Figure 1.

**Figure 2:**
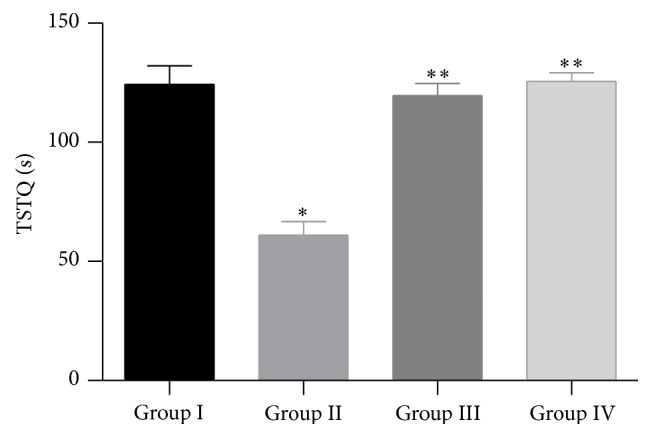
Effect of ethanol extract of leaves of* Malva parviflora* on escape time spent in target quadrant (TSTQ) in Morris water maze test. The values are mean ± SEM. *n* = 10. ^*^ *P* < 0.01, significant difference when compared to normal control mice. ^**^ *P* < 0.01, significant difference when compared to Alzheimer group mice (One-way ANOVA followed by Tukey's post hoc test).

In the Morris water maze test, the results of time spent in the target quadrant (TSTQ) show that there was a significant decrease in time spent in the target quadrant (TSTQ) of Group II (Alzheimer group) mice when compared to Group I (Normal control) mice (*P* < 0.01). However, time spent in the target quadrant (TSTQ) was increased significantly in Group III (Treatment group; 250 mg/kg) and Group IV (Treatment group; 500 mg/kg) mice when compared to Group II (Alzheimer group) mice (*P* < 0.01) Figure 2.

There was a significant reduction in the glutathione peroxidase, glutathione reductase, catalase, and SOD levels of Group II (Alzheimer group) mice when compared to Group I (Normal control) mice (*P* < 0.01). The levels of aforesaid enzymes were increased significantly in Group III (Treatment group; 250 mg/kg) and Group IV (Treatment group; 500 mg/kg) mice when compared to Group II (Alzheimer group) mice (*P* < 0.01). However, there was a significant increase in lipid peroxidase levels of Group II (Alzheimer group) mice when compared to Group I (Normal control) mice (*P* < 0.01). The levels of lipid peroxidase were decreased significantly in Group III (Treatment group; 250 mg/kg) and Group IV (Treatment group; 500 mg/kg) mice when compared to Group II (Alzheimer group) mice (*P* < 0.01) Table 1.

## 5. Discussion

In the present study, ethanol extract of the leaves of* Malva parviflora* (250 and 500 mg/kg, p.o.) has shown a significant neuroprotective effect in mice. To the best of our knowledge, this is the first study showing neuroprotective activity of* Malva parviflora*. The Morris water maze model was used as a behavioural model to assess the effect of the extract on memory. The Morris water maze model is widely used to assess the effects of drugs on learning and memory. In this model, a decrease in escape latency (EL) and an increase in time spent in the target quadrant (TSTQ) indicate improvement of learning and memory [16]. The extract of leaves of* Malva parviflora *showed a significant decrease in EL and a significant increase in TSTQ in A*β* injected mice.

The extract of* Malva parviflora *leaves also showed positive effects on mouse brain. In our study, ICV injection of A*β* (25–35) increased the levels of lipid peroxidation as indicated by increased levels of lipid peroxidase. Increase in lipid peroxidase indicates the increased levels of reactive oxygen species (ROS) because lipid peroxidation (LPO) is catalysed by ROS. LPO may become very harmful due to the fact that a single-free radical can cause damage to a number of polyunsaturated fatty acid (PUFA) molecules. Oxidized polyunsaturated fatty acid (PUFA) can produce more neurotoxic molecules such as malondialdehyde (MDA), 4 hydroxynonenal (HNE), and acrolein [22, 23]. The brain is specifically vulnerable to oxidative stress and lipid peroxidation. Highly efficient mechanisms are needed to protect nerve cells because they have very restricted regeneration capacity. Chain-braking antioxidants like vitamin E have the capability of terminating the LPO chain reaction [24].

The results of our study show that after ICV injection of A*β* (25–35) the activity of SOD was decreased in brain which could be a result of inactivation of SOD by H_2_O_2_. This result intimated a compensatory response to oxidative stress due to an increase in the synthesis of endogenous H_2_O_2_. Therefore, the elevation in the level of SOD in ethanol extract treated animals foretells that* Malva parviflora *may possess free radical scavenging potential, which could be beneficial against the pathological alterations produced by the presence of O_2_^•−^ and OH [25].

One of the most important antioxidants in mammalian cells is glutathione (GSH). This nonprotein thiol antioxidant is the major intracellular redox buffer. GSH-dependent detoxification involves glutathione peroxidase (GSHx), which has a pivotal role in the elimination of hydrogen and organic peroxides and leads to the formation of oxidized glutathione but is reduced back to its thiol form (GSH) by glutathione reductase (GSHr), leading to the consumption of NADPH, which is chiefly formed by pentose phosphate pathway. GSH is involved in xenobiotic conjugation with the help of different glutathione S-transferase isoenzymes. The inhibition of GSH synthesis causes an increase in amyloid-*β*- (A*β*-) mediated cell death and intracellular A*β* accumulation [26].

Various clinical trials have reported strong evidences that impairment in memory observed in rodents is linked with the altered levels of GSH in the brain and with the activities of antioxidant enzymes [27]. Elevation of brain oxidative status of amnesic rodents was similar as observed in the clinical pathology of Alzheimer's disease patients [28]. Amyloid-*β*- (A*β*-) mediated increase in LPO and decrease in glutathione (GSH), a natural antioxidant, are one of the principal causes of neurodegeneration [29].

In our study, amyloid-*β* (A*β*) administration caused a significant decrease in the activity of glutathione peroxidase (GSHx), glutathione reductase (GSHr), catalase, and superoxide dismutase (SOD) in Alzheimer group mice. The administration of ethanol extract of* Malva parviflora *leaves restored the levels of aforesaid enzymes in A*β* injected mice to similar levels as seen in normal control mice. So, we can conclude that the extract possesses the neuroprotective activity in mice. The neuroprotective activity of* Malva parviflora *can be attributed to the presence of flavonoids and polyphenolic compounds in the leaves of the plant [30]. Therefore, in future, we intend to conduct studies on isolated compounds of the leaves of this plant.

## 6. Conclusion

The present study has shown significant neuroprotective effect of ethanol extract of the leaves of* Malva parviflora* at 250 and 500 mg/kg doses in mice. To the best of our knowledge, this is the first study on the neuroprotective activity of the leaves of* Malva parviflora*.

## Figures and Tables

**Table 1:** Effect of ethanol extract of leaves of* Malva parviflora* on enzymes of mouse brain.

Enzymes	Group I	Group II	Group III	Group IV
Glutathione peroxidase (GSHx)	109.7 ± 5.3	63.8 ± 6.2^*^	106.3 ± 5.3^**^	107.0 ± 8.4^**^
(nmol GSH oxidized/mg protein)				
Glutathione reductase (GSHr)	6.2 ± 1.03	2.4 ± 0.6^*^	5.5 ± 0.8^**^	6.8 ± 0.6^**^
(NADPH oxidized/min/mg protein)				
Catalase	58.1 ± 0.71	37.3 ± 0.6^*^	57.2 ± 0.9^**^	58.8 ± 0.4^**^
(nmol H_2_O_2_ decomposed/min/min protein)				
Superoxide dismutase	6.7 ± 0.73	2.7 ± 0.3^*^	6.1 ± 0.6^**^	6.2 ± 0.5^**^
(Units/min/mg protein)				
Lipid peroxidase	2.4 ± 0.1	4.3 ± 0.2^*^	2.0 ± 0.3^**^	2.3 ± 0.4^**^
(nmol MDA/mg protein)				

The values are mean ± SEM. *n* = 10.

^*^ *P*, significant difference when compared to normal control mice.

^**^ *P*, significant difference when compared to Alzheimer group mice (One-way ANOVA followed by Tukey's post hoc test).
